# Association between specific social activities and depressive symptoms among older adults: A study of urban-rural differences in China

**DOI:** 10.3389/fpubh.2023.1099260

**Published:** 2023-03-23

**Authors:** Tanqian Han, Mei Han, Paulo Moreira, Hongxia Song, Ping Li, Zhenlong Zhang

**Affiliations:** ^1^Department of Nursing, The First Affiliated Hospital of Shandong First Medical University & Shandong Provincial Qianfoshan Hospital, Jinan, China; ^2^School of Nursing, Shandong First Medical University & Shandong Academy of Medical Sciences, Taian, China; ^3^International Healthcare Management Research and Development Centre, Shandong Provincial Qianfoshan Hospital, Jinan, Shandong, China; ^4^Atlantica Instituto Universitario, Gestao em Saude, Oeiras, Portugal

**Keywords:** elderly adults, social activities, depressive symptoms, urban-rural differences, CHARLS (wave4)

## Abstract

**Background:**

Engaging in social activities can help older persons with their depressed symptoms. Few studies, however, have looked into the connection between social interactions and depressed symptoms in Chinese older persons. The aim of this study was to investigate differences in older Chinese individuals' social activity involvement and depressive symptoms across urban and rural settings.

**Methods:**

A cross-sectional investigation using information from the 2018 China Health and Retirement Longitudinal Study (CHARLS), which was limited to older individuals aged 60 and over. Generalized linear models were constructed to assess the effects of participants' characteristics and specific social activities on CES-D scores. The association between specific social activities and depressed symptoms was investigated using multivariate logistic regression analysis.

**Results:**

In this study, it was discovered that older individuals had a prevalence of depressed symptoms of 36.2%, with rural older adults having a greater prevalence of depressive symptoms (39.7%) than urban older adults (30.9%). Our results showed that for urban respondents, providing help to others (not regularly. *OR* = 0.753, 95% *CI*: 0.579–0.980, *P* = 0.035), going to a sport (not regularly. *OR* = 0.685, 95% *CI*: 0.508–0.924, *P* = 0.013), and using the Internet (not regular. *OR* = 0.613, 95% *CI*: 0.477–0.789, *P* < 0.001; almost weekly. *OR* = 0.196, 95% *CI*: 0.060–0.645, *P* = 0.007) were all significantly and negatively associated with depressive symptoms, while for rural respondents, interacting with friends (not regularly. *OR* = 1.205, 95% *CI*: 1.028–01.412, *P* = 0.021) and using the Internet (not regularly. *OR* = 0.441, 95% *CI*: 0.278–0.698, *P* < 0.001) were significantly and negatively associated with depressive symptoms.

**Conclusions:**

According to our research, there is a cross-sectional relationship between participating in a specific social activity and depressed symptoms in Chinese older adults, and this relationship varies across urban and rural older adults. This suggests that taking part in specific social activities may be crucial for reducing depression symptoms in older persons, developing more focused interventions that might support healthy aging, and offering a guide for policymakers and activists working to improve the mental health of seniors.

## 1. Introduction

Depression is a mood disorder characterized by a persistent feeling of sadness and/or inability to experience joy, accompanied by daily functional impairment (1). Projections by the World Health Organization suggest that it will be the most common disabling disease in the world by 2030 (2). Being one of the most prevalent illnesses affecting the elderly, it has emerged as a serious global public health issue (3). China is becoming a rapidly aging country, with data from the National Bureau of Statistics showing 264 million people aged 60 years and older (18.70%) by the end of 2020, and predicting that China will have one of the highest proportions of older people in the world (4). Depression not only worsens older citizens' health, but also lowers the standard of living for both individuals and their families. Age-related depression in older persons must be prevented and treated early.

Participation in social activities is a key element of successful aging and can help alleviate depressive symptoms in older adults. Studies from around the world have looked into the link between social engagement and health in elderly people. For instance, Croezen (5) discovered that the type of social activity had an impact on how strongly depressive symptoms were related to social involvement in older persons in 10 different European countries. Sibalija et al. (6) found that among older adults in Canada, positive social interactions and lower depression ratings were correlated with social involvement. According to Enunsoo's study, older Korean adults who participated in more varied and regular activities experienced a further reduction in the probability of depressive symptoms (7). Furthermore, a study from National Trends in Health and Aging found that online social engagement may moderate the negative effects of other risk factors on psychosocial outcomes (8). Nevertheless, Chinese older folks may choose their social activities differently as a result of China's distinctive culture and social system. For instance, family bonds are highly valued in Chinese culture, which has a substantial impact on depression symptom (9). And according to certain research, China has a lower rate of senior citizens participating in social activities than other nations (10, 11). Additionally, compared to other nations, China currently lacks a lot of mental health resources and support networks, particularly in the areas of professional counseling and early intervention (12). Therefore, in order to create more targeted interventions that might support healthy aging and give government policy makers and advocates better information to improve the mental health of older adults and reduce the prevalence of depression in China, it is imperative to look into the relationship between social interactions and depressive symptoms in older people in that country. Recent research in China confirms these challenges as related to the Emotional Support Mechanism of the Mental Health of Empty Nesters (13).

Due to China's increasing urbanization, the difference in socioeconomic development between urban and rural areas has had an impact on the prevalence of depressive symptoms among older people in those locations (14). According to earlier research, older people in rural areas had an estimated 1.88 times higher frequency of depression than older people in urban areas., and 63.8% of older adults in urban areas in China are socially active compared to 45.4% in rural areas (15). Consequently, while examining the connection between social activity and depression among older persons in China, it is important to take into account the disparities between urban and rural locations. Moreover, participation in social activities is strongly correlated with activity frequency (16). Studies have shown that social activity participation at an appropriate frequency can help improve the mental health of older adults (17, 18). There has, however, been little discussion regarding the optimal frequency and type of activity, as well as the target group and their sociocultural background, despite the fact that earlier research have shown significant evidence for the prevention of depression. In order to better understand how social activity involvement and depression symptoms in Chinese older persons relate to one another, this study focused on the disparities between urban and rural areas and put forward the following three hypotheses.

Hypothesis 1: Older adults in urban and rural areas have significantly different prevalence rates for depressive symptoms. The prevalence of depressive symptoms was higher among older people in rural areas than in urban ones.

Hypothesis 2: The depressive symptoms of the elderly in urban and rural areas are affected by different factors. Although the same influencing factors apply to urban and rural older adults, both affect depressive symptoms to different extents.

Hypothesis 3: The impact of different types and frequencies of social activities on the depression of the elderly is also different between urban and rural areas.

## 2. Methods

### 2.1. Data and participants

The 2018 China Longitudinal Study on Health and Retirement (CHARLS) was conducted by the National School of Development of Peking University based on multi-level sampling, which provided the data for the current study. The 20,813 residents in all, representing 11,535 households, participated in the survey. Face-to-face interviews were used to gather thorough participant data. Large-scale, extensive, and highly representative, CHARLS is a longitudinal study. It offers a thorough analysis of the health state and way of life of Chinese older individuals in retirement, including their physical and mental wellbeing, dietary intake, and household expenses. Additionally, the CHARLS is able to follow subjects into their post-retirement years, offering a reliable data source for the retirement security system for Chinese older persons. A more detailed CHARLS survey design has been described elsewhere (19).

Apply for a CHARLS data usage license from the National School of Development, Peking University. Prior to taking the survey, each participant supplied their informed consent for inclusion. Finally, 9,478 study participants were enrolled, from which the following sample data were omitted: (i) age < 60 years; (ii) have been diagnosed by a doctor with a memory-related disorder and severe psychiatric disorders other than depression; (iii) missing data of social activities; (iv) not completing the Center for Epidemiological Studies Depression Scale (CES-D 10); (v) missing and illogical data in important fields.

### 2.2. Assessment of specific social activities

The CHARLS questionnaire evaluated specific social activities, such as “interacting with friends,” “playing Mahjong, chess, cards or going to community club,” “providing help to family, friends, or neighbors who do not live with you for free,” “going to a sport,” “taking part in a community-related organization,” “doing voluntary or charity work,” “caring for a sick or disabled adult who does not live with you,” “attending a course,” “stock investment,” and “using the Internet.” Interviewees were asked whether they had engaged in any of the listed social activities in the past month. If they answered “yes,” they were further asked the frequency of their social activity participation in the past month. The answers were categorized as “not regularly,” “almost every week,” or “almost daily.” Activities for which they answered “no” more than 95% of the time will be excluded to ensure that these activities have considerable acceptability within the studied population. In the Supplementary material, the excluded activities are described in great depth (Supplementary Table 1). Four activities were ultimately chosen for this study's independent variables, respectively “interacting with friends,” “providing help to family, friends or neighbors,” “playing Ma-Jong and other games,” “going to a sport,” and “used the Internet.”

### 2.3. Assessment of depressive symptoms

A condensed version of the Center for Epidemiology Studies Depression Scale (CES-D) is used for the depressive symptoms survey in the CHARLS project, which was developed by the American Center for Epidemiological Survey and is an extensive Depressive symptom measurement tool used. The scale contains 10 symptom items, each of which is scored on a 4-point scale, 0 = rarely or not at all (< 1d); 1 = not too much (12d); 2 = sometimes or half the time (3–4d); 3 = most of the time (5–7d). The items “I am hopeful for the future” and “I am very happy” are negatively scored, with a total score of 0 to 30. A total score of ≥10 was defined as depressive symptoms, and a score of < 10 was defined as normal.

### 2.4. Covariates

Demographic characteristics, health status, and lifestyle factors were considered as covariates in this study. Demographic characteristics included age (years), gender (female and male), residence (urban and rural), highest education (illiterate, elementary and below, junior high and above), and marital status (married with spouse or not). Health status included self-reported health status (poor, fair or good), the number of chronic diseases (none, one, or two or more). Lifestyle factors included sleep duration (< 6, 6–8,>8 h), alcohol consumption (drinking more than once a month, drinking but less than once a month or none of these).

### 2.5. Statistical analysis

Using R statistical software 4.2.1, all data were examined. Continuous data mean and standard deviation, classification data percentage and other indicators are used to describe the overall characteristics of the respondents. For comparisons of continuous and categorical variables, respectively, independent *t*-tests and the χ*2* test were used. Under different types and frequencies of social activities, the prevalence of depressed symptoms was examined using the χ*2* test for various types and frequency of social activity. Generalized linear models were constructed to assess the effects of respondent characteristics and specific social activities on CES-D scores, with attention to urban-rural differences. Multiple logistic regression analysis was used to examine the relationship between specific social activities and depressive symptoms, and to compare urban-rural differences in different types and frequencies of social activities. The difference is statistically significant if *P* < 0.05.

## 3. Results

### 3.1. Sample characteristics

Table 1 displays the participant characteristics. A total of 9,424 individuals were included in the study. The mean age of the participants was 68.54years (standard deviation [SD] = 6.54). Of the participants, 4,677(49.6%) were male and 4,747 (50.4%) were female; 3,716 (39.4%) lived in urban areas while 5,708 (60.6%) lived in rural areas; 79.5% were married or cohabiting and 20.5% lived alone, and more than half of the participants were uneducated. Only 30.2% of the participants self-reported good health status, 63.7% had two or more chronic diseases, more than 50% sleeping 6 to 8 h, and 68% did not drink alcohol. In the sample, 36.2% of people reported having depressed symptoms (30.9% in urban areas; 39.7% in rural areas). Statistically significant variations in the prevalence of depression were detected, according to gender, place of residence, education level, marital status, self-reported health status, number of chronic diseases, sleep duration, and alcohol consumption were statistically significant (*P* < 0.001).

**Table 1:** The basic characteristics of the study population, *n* (%).

**Characteristics**	**Total sample**	**Depressive symptoms**		**χ^2^/*t***	***P***
	**(*****n*** = **9,424)**	**No**	**Yes**		
Age (years)	68.54 ± 6.54	68.61 ± 6.58	68.43 ± 6.47	1.305	0.192
Gender				175.251	< 0.001
Male	4,677 (49.6)	3,292 (70.4)	1,385 (29.6)		
Female	4,747 (50.4)	2,719 (57.3)	2,028 (42.7)		
Residence				74.484	< 0.001
Urban	3,716 (39.4)	2,567 (69.1)	1,149 (30.9)		
Rural	5,708 (60.6)	3,444 (60.3)	2,264 (39.7)		
Highest education				114.046	< 0.001
Illiterate	2,799 (29.7)	1,607 (57.5)	1,187 (42.5)		
Elementary and below	4,245 (45.0)	2,691 (63.4)	1,553 (36.6)		
Junior high and above	2,386 (25.3)	1,713 (71.8)	673 (28.2)		
Marital status				34.678	< 0.001
Married with spouse	7,491 (79.5)	4,889 (65.3)	2,602 (34.7)		
No spouse	1,933 (20.5)	1,122 (58.0)	811 (42.0)		
Self-reported health status				571.564	< 0.001
Good	2,010 (21.3)	1,575 (78.4)	435 (21.6)		
Fair	4,569 (48.5)	3,103 (67.9)	1,466 (32.1)		
Poor	2,845 (30.2)	1,333 (46.9)	1,512 (53.1)		
Number of chronic diseases				240.028	< 0.001
0	1,338 (14.2)	1,042 (70.9)	296 (22.1)		
1	2,079 (22.1)	1,472 (70.8)	607 (29.2)		
≥2	6,007 (63.7)	3,497 (58.2)	2,510 (41.8)		
Sleep duration (h)				339.044	< 0.001
< 6	3,585 (38.0)	1,871 (52.2)	1,714 (47.8)		
6–8	4,846 (51.4)	3,457 (71.3)	1,389 (28.7)		
>8	993 (10.5)	683 (68.8)	310 (31.2)		
Alcohol consumption				70.582	< 0.001
Drink more than once a month	2,382 (25.3)	1,681 (70.6)	701 (29.4)		
Drink but less than once a month	636 (6.7)	422 (66.4)	214 (33.6)		
None of these	6,405 (68.0)	3,908 (61.0)	2,497 (39.0)		

### 3.2. Prevalence of depression symptoms in various social activity types and frequency

As shown in Table 2, 30.2% interacted with friends, 18.4% provided help to family, friends, or neighbors, 10.8% played mahjong and other games, 8.3% took part in a sport, and 7.9% used the Internet, within the overall participants without depressive symptoms. In comparison to older individuals in rural areas, more older adults in urban areas participated in the aforementioned five social activities (*P* < 0.05). Among overall participants and urban older adults, there were significant differences in the prevalence of depressive symptoms for three specific types of social activities: providing help to family, friends, or neighbors, participating in sports, and using the Internet. Only one particular social activity—using the Internet—demonstrated a substantial difference in the occurrence of depressive symptoms across elderly residents of rural areas.

**Table 2:** Prevalence of depressive symptoms in different type and frequency of social activity participation.

**Type and frequency of social activities**	**Total sample (%)**		***χ^2^***	***P***	**Urban (%)**		***χ^2^***	***P***	**Rural (%)**		***χ^2^***	***P***
	**No**	**Yes**			**No**	**Yes**			**No**	**Yes**		
Interacting with friends			1.039	0.792			2.450	0.484			6.182	0.103
No participation	69.8	68.8			67.0	68.8			71.9	68.8		
Not regularly	13.6	13.9			14.2	12.4			13.2	14.7		
Almost every week	6.2	6.6			6.8	6.7			5.8	6.5		
Almost daily	10.3	10.7			12.0	12.1			9.1	9.9		
Providing help to family, friends or neighbors			17.880	**< 0.001**			10.482	**0.015**			4.04	0.257
No participation	81.6	85			76.3	80.9			85.6	87.1		
Not regularly	6.9	5.7			9.7	7.4			4.8	4.8		
Almost every week	5.5	4.5			7.2	6.3			4.2	3.5		
Almost daily	6	4.8			6.9	5.4			5.4	4.5		
Playing Ma-jong and other games			1.494	0.684			2.737	0.434			3.397	0.334
No participation	89.2	89.9			88.6	90.0			89.6	89.9		
Not regularly	1.2	1.1			1.2	1.4			1.2	0.9		
Almost every week	2	1.7			2.3	2.1			1.7	1.5		
Almost daily	7.7	7.3			7.9	6.5			7.5	7.7		
Going to a sport			14.670	**0.002**			11.780	**0.008**			1.246	0.742
No participation	93.7	95.5			89.6	92.9			96.8	96.8		
Not regularly	4.8	3.2			8.5	5.4			2.0	2.0		
Almost every week	0.7	0.6			0.8	1.0			0.6	0.4		
Almost daily	0.6	0.7			1.1	0.8			0.6	0.7		
Used the internet			61.231	**< 0.001**			27.355	**< 0.001**			14.308	**0.003**
No participation	92.1	96.1			85.5	91.3			97.1	98.6		
Not regularly	6.7	3.3			12.5	7.8			2.4	1.1		
Almost every week	0.6	0.1			1.2	0.3			0.2	0.1		
Almost daily	0.5	0.4			0.7	0.6			0.3	0.3		

The bold values indicate that the *P* values are below 0.05 and are statistically significant.

### 3.3. Associations between CES-D scores and effect factors and urban-rural differences

As shown in Table 3, the results of the generalized linear model indicated that women (β = 1.120, *CI* = 0.866–1.374), no spouse (β = 0.524, *CI* = 0.243–0.805), self-reported fair health (β = 0.595, *CI* = 0.311–0.878), self-reported poor health (β = 2.696, *CI* = 2.368–3.023), and having one (β = 0.596, *CI* = 0.231–0.960) or two or more chronic diseases (β = 1.469, *CI* = 1.138–1.799) were associated with higher CES-D scores (*P* < 0.05). In addition, elementary education and below (β = −0.436, *CI* = −0.711–1.164), high school education and above (β = −0.565, *CI* = −0.853–−0.278), 6–8 h sleep (β = −1.874, *CI* = −2.107–−1.641), and >8 h sleep (β = −1.983, *CI* = −2.357–−1.609) was associated with lower CES-D scores (*P* < 0.05). Overall depression scores were significantly higher in rural compared to urban older adults. Primary and lower secondary education, compared to illiteracy, were significantly inversely related to depressed symptom ratings in urban aged adults, who made up the majority of the population (*P* < 0.05). The tendency for higher depressive symptom scores among older adults with average self-reported health status relative to good self-reported health status was only found among urban older adults (*P* < 0.05).

**Table 3:** Associations between CES-D scores and effect factors and urban-rural differences.

**Characteristics**	**Total sample**					**Urban**					**Rural**				
	***B***	***SE***	***95%CI***		***P***	***B***	***SE***	***95%CI***		***P***	***B***	***SE***	***95%CI***		***P***
Age (years)	−0.061	0.009	−0.079	−0.044	**<0.001**	−0.029	0.012	−0.053	−0.005	**0.019**	−0.091	0.012	−0.115	−0.067	**<0.001**
**Gender**															
Male					1.000			1.000					1.000		
Female	1.12	0.130	0.866	1.374	**<0.001**	0.868	0.182	0.511	1.225	**<0.001**	1.056	0.178	0.707	1.405	**<0.001**
**Highest education**															
Illiterate					1.000			1.000					1.000		
Elementary and below	−0.436	0.140	−0.711	1.164	**0.002**	−0.600	0.203	−0.997	−0.203	**0.003**	−0.144	0.193	−0.522	0.233	0.454
Junior high and above	−0.565	0.147	−0.853	−0.278	**<0.001**	−0.532	0.196	−0.916	−0.148	**0.007**	−0.378	0.220	−0.81	0.054	0.087
**Marital status**															
Married with spouse					1.000			1.000					1.000		
No spouse	0.524	0.143	0.243	0.805	**<0.001**	0.387	0.205	−0.016	0.789	0.06	0.586	0.195	0.203	0.969	0.003
Self–reported Health Status															
Good					1.000			1.000					1.000		
Fair	0.595	0.145	0.311	0.878	**<0.001**	0.989	0.196	0.604	1.374	**<0.001**	0.345	0.204	−0.055	0.746	0.091
Poor	2.696	0.167	2.368	3.023	**<0.001**	2.798	0.242	2.323	3.273	**<0.001**	2.565	0.227	2.120	3.010	**<0.001**
**Number of chronic diseases**															
0					1.000			1.000					1.000		
1	0.596	0.186	0.231	0.960	**0.001**	0.823	0.272	0.29	1.356	**0.002**	0.507	0.249	0.02	0.995	**0.041**
≥2	1.469	0.169	1.138	1.799	**<0.001**	1.296	0.242	0.822	1.769	**<0.001**	1.591	0.229	1.143	2.039	**<0.001**
**Sleep duration (h)**															
< 6					1.000			1.000					1.000		
6–8	−1.874	0.119	−2.107	−1.641	**<0.001**	−1.957	0.168	−2.285	−1.628	**<0.001**	−1.809	0.163	−2.129	−1.489	**<0.001**
>8	−1.983	0.191	−2.357	−1.609	**<0.001**	−1.888	0.308	−2.490	−1.286	**<0.001**	−2.084	0.244	−2.562	−1.606	**<0.001**
**Alcohol consumption**															
Drink more than once a month					1.000			1.000					1.000		
Drink but less than once a month	0.177	0.237	−0.288	0.641	0.351	0.131	0.311	−0.477	0.740	0.672	0.262	0.345	−0.414	0.939	0.447
None of these	0.051	0.140	−0.224	0.326	0.637	−0.07	0.204	−0.471	0.330	0.731	0.122	0.189	−0.248	0.492	0.517

The bold values indicate that the *P* values are below 0.05 and are statistically significant.

### 3.4. Association of different types and frequencies of social activities with depressive symptoms and urban-rural differences

**Figure 1:**
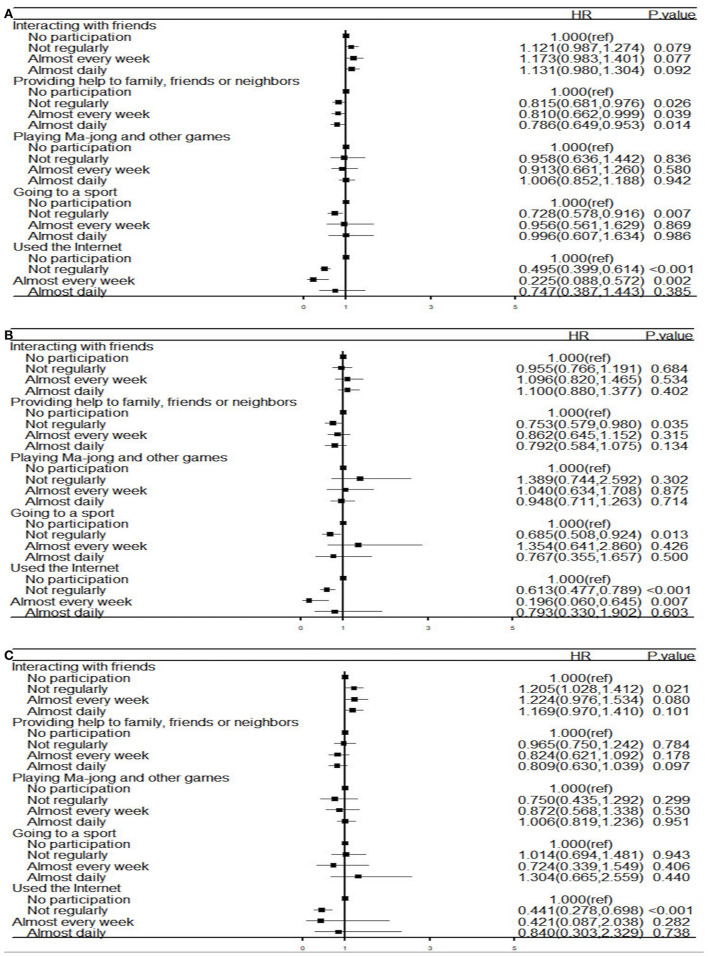
Forest plot of association between risk factors and depression **(A)** the entire study population (*n* = 9,9424); **(B)** the urban (*n* = 3,716); **(C)** the rural (*n* = 5,708). HR, hazard ratios; Analysis of multiple logistic regression models.

Figure 1 shows that for urban respondents, providing help to others (not regularly. *OR* = 0.753, 95% *CI*: 0.579–0.980, *P* = 0.035), going to a sport (not regularly. *OR* = 0.685, 95% *CI*: 0.508–0.924, *P* = 0.013), and using the Internet (not regular. *OR* = 0.613, 95% *CI*: 0.477–0.789, *P* < 0.001; almost weekly. *OR* = 0.196, 95% *CI*: 0.060–0.645, *P* = 0.007) were all significantly and negatively associated with depressive symptoms, while for rural respondents, interacting with friends (not regularly. *OR* = 1.205, 95% *CI*: 1.028–01.412, *P* = 0.021) and using the Internet (not regularly. *OR* = 0.441, 95% *CI*: 0.278–0.698, *P* < 0.001) were significantly and negatively associated with depressive symptoms. In the sensitivity analysis, we found no discernible differences in the generalized linear model's findings (Supplementary Table 2).

## 4. Discussions

### 4.1. Risk factors affecting depressive symptoms in the elderly and urban-rural differences

According to our research, there is a considerable variation in the prevalence of depressive symptoms among elderly people in urban and rural areas. Compared with urban elderly, the prevalence of depressive symptoms in rural elderly is significantly higher, which is consistent with Wang et al. (20), which answered Hypothesis 1 (Older adults in urban and rural areas have significantly different prevalence rates for depressive symptoms.). The disparities between old Chinese urban and rural populations in terms of economic standing and cognitive reserve may be responsible for this. Li (21) reports that rural seniors in rural China may face stress associated with relatively low incomes. The rural economy is underdeveloped, and the elderly generally have no fixed income and less access to medical services and social support. Urban older individuals, on the other hand, typically have favorable economic circumstances. So, it needs to deepen the reform to solve the problem of inequality between urban and rural areas in China, as argued by Zheng in a recent study discussing the development of Rural healthcare Systems (22). This study found that in urban and rural areas, the prevalence of depressive symptoms in the elderly was significantly different due to sex, marital status, education level, self-reported health status, number of chronic diseases, sleep duration and drinking frequency, which answered Hypothesis 2 (The depressive symptoms of the elderly in urban and rural areas are affected by different factors). In accordance with the study's findings, women experience depressive symptoms more frequently than men do, which is in line with findings from earlier research (4, 23). This may be related to the traditionally lower socioeconomic status of women and their lower sense of self-worth and self-identity. Thus, we should concentrate on the mental health issues that affect elderly women, provide them with greater assistance and care in the avoidance of family division of labor, social support, and other areas, and motivate them to get out and engage in more social activities and communication.

For urban older adults, educational status was one of the significant factors relating to depressive symptoms, but there was no significant effect on depressive symptoms in rural older adults. The reason for this may be the generally low level of education in rural areas and the absence of large differences in overall educational attainment. For urban respondents, the more educational opportunities they had, the more likely they were to have higher levels of cognition and better self-regulation. In our study, in urban areas, the prevalence of depression was 3.273 times higher among older adults with poor health than those with good health, and 1.374 times higher among those with fair health than those with good health, which is similar to existing findings (24, 25). The more optimistic one is about their health, the more likely they are to take a positive outlook on life, and the lower their risk of developing depressive symptoms, the better their self-reported health condition is Kim et al. (26). A significant contributing factor to depression symptoms in older persons is chronic disease. In urban areas, the risk of depressive symptoms was 1.769 times higher among older adults with ≥2 chronic diseases than without any chronic disease and 1.143 times higher in rural areas, with little urban-rural difference. This is also supported by previous studies (27, 28). Most chronic diseases have a long course and may even require lifelong treatment, affecting their quality of life and increasing the financial burden on families, which may trigger and exacerbate the onset and progression of depression (29). Furthermore, this study found that sleep duration 6–8 h and >8 h were protective factors against depressive symptoms in older adults in both urban and rural areas, relative to sleep duration < 6 h. It has been confirmed that adjusting sleep duration decreases the incidence of depressive symptoms (30). Consequently, sleep duration should be appropriately adjusted to improve the mental health of middle-aged and elderly people, and in turn delay and prevent the occurrence of depression.

### 4.2. Association of specific social activities and their frequency on depressive symptoms in older adults and urban-rural differences

Our study's findings supported earlier research by demonstrating that older persons in rural areas participated in social activities at a considerably lower rate than older adults in urban areas (31). The unequal socioeconomic growth and resource distribution between urban and rural areas may be to blame for this. Rural areas lack adequate human and material resources to help older individuals in participating in social activities, and their service infrastructure for older adults is generally underdeveloped (32). It has been shown that the frequency of older adults' participation in social activities depends on the ease of access to activity centers (33). As such, government policy makers and leaders should improve the disparity in resource allocation between urban and rural areas so that people have equal access to public resources and opportunities to participate in social activities. Moreover, the results of logistic regression in this study showed that for urban respondents, providing help to others, participating in sports activities, and using the Internet were all significantly and negatively associated with depressive symptoms, while for rural respondents, interacting with friends and using the Internet were strongly and negatively linked with depressed symptoms, which answered Hypothesis 3 (The impact of different types and frequencies of social activities on the depression of the elderly is also different between urban and rural areas).

In contrast to older individuals in rural areas, urban older adults had a significantly and adversely correlated relationship with depression symptoms when it came to not routinely helping family, friends, or neighbors, which is slightly different from other relevant investigations (5, 7, 34), is generally in agreement with the work by G. Cheng et al. (35). The explanation for this may be that urban older adults have a stronger internal drive to engage in social interactions than rural older adults, actively choosing to offer help to others themselves to enhance social interactions and increase their sense of self-worth. Kim and Jung (36) surveyed that for older adults living alone, regular monitoring and interventions for social interactions are important, and according to Fancourt et al. (37), older persons who participated in cultural activities once every few months or more had a 48% reduced risk of depression compared to those who did not, and those who participated once a month or more had a 32% lower risk. Hence, while taking action to reduce depression symptoms in older persons, it is important to take into account how frequently people engage in social connections.

According to the results of the current study, irregular physical activity is significantly and negatively linked with depression symptoms in older urban individuals but not in older rural adults. This result is somewhat in line with a research on urban elderly persons in China (38, 39). The possible reason for this situation is that most rural residents are farmers who spend considerable time in labor (the intense physical activity) and they may be less interested in participating in other sports or social clubs. There is less widespread availability of sports and social club activities among rural residents compared to urban residents. A cohort study from Korea indicated that sedentary status was associated with depressive symptoms in a *U*-shaped correlation and that maintaining proper physical activity during the year was beneficial in reducing episodic depressive symptoms (38). In addition, for its effects some studies have suggested that the potential active components of participation in physical activity may trigger causal mechanisms to reduce depression (40). The provision of social resources, for instance, is one of the active elements of clubs. These resources can help people feel less lonely and can be utilized to help people form social identities (41). Therefore, government directors to prevent the occurrence of depressive symptoms in older adults should promote appropriate physical activity and better frequency of physical activity.

Based on the study's findings, irregular social engagement with friends was substantially and negatively correlated with depression symptoms in older individuals in rural areas but not in urban areas (42). This may be due to differences in interpersonal networks resulting from differences in lifestyles in rural and urban areas to China. Compared to urban older adults, social networks in rural China are limited with essentially traditional kinship relationships, but which are strong and stable. Wei et al. (13) and Domènech-Abella et al. (43) argued that the social networks of urban residents consist of weak ties among people from different backgrounds with lower levels of intimacy. Cheng et al. (44) revealed that weaker social networks, which were associated with more sleep disorders, were more likely to cause depressive emotions. As a result, the state of older persons' mental health must be taken into consideration in rural areas with reduced interaction with friends to prevent their depressive symptoms and this concern is also linked with the current international debate on the Ethics of Health Promotion (45).

This study also found that irregular Internet use was substantially and negatively linked with depressed symptoms in urban older individuals, but irregular Internet use was just negatively associated with depressive symptoms among older adults in rural areas. This is different from other Chinese research (34, 46). This may be related to the gap in Internet coverage between urban and rural China. The existing computer technology and the use of the Internet have acted as an essential modern form of leisure activities, especially the emergence of short videos on mobile phones, which are popular among the public at large. For older adults, using the Internet makes it easier to befriend new people, improve the quality of communication with others, and enrich their spiritual life, which reduces their sense of loneliness (43). In addition, more and more psychological interventions, cognitive-behavioral therapies are conducted through the Internet (47). In future studies, we should pay more attention to personalized psychological interventions and guidance through the Internet. Playing mahjong and other games was also included as a social activity with Chinese characteristics, and we investigated the variations in the incidence of depressive symptoms in older persons among their frequencies. Regrettably, we were unable to detect a difference, and the disparities between urban and rural areas were also not significant. Further study is required on this subject because previous Chinese researchers have discovered notable variations in cognitive performance between older persons who played mahjong and other games (48).

### 4.3. Strengths and limitations

In this study, we enriched the urban-rural differences in the factors influencing depressive symptoms in older adults in China and explored the effects of specific social activities and their frequencies on depressive symptoms in older adults and assessed their urban-rural differences for comparison. In addition, this study involved a large number of participants and assessed multiple influencing factors with the aim of identifying high-risk groups and finding targeted interventions to reduce the prevalence of depression in older adults and improve quality of life. However, there are a few limitations in this study. First off, because the research was cross-sectional, it was not able to investigate the causal relationships between social interactions and depressed symptoms in older adults. Depression symptoms among the participants would have made it challenging for them to partake in social activities. Second, the study depended on self-reports, which would make it more likely that some respondents gave false or inaccurate information, leading to recall bias. Third, the evaluation of the “socializing with friends” activity can be off because it might overlap with other activities. As an illustration, playing sports with friends is an option. Finally, the CHARLS was unable to evaluate the standard of interpersonal interactions that took place during social activities; this should be taken into account in future research.

## 5. Conclusion

According to our research, there is a cross-sectional relationship between participating in a specific social activity and depressed symptoms in Chinese older adults, and this relationship varies across urban and rural older adults. This suggests that taking part in social activities may be crucial for easing depression symptoms in older persons, offering a guide for policymakers and activists working to improve the mental health of seniors. This study may lead to new treatments and prevention strategies for elderly depression, as well as a relatively lower risk of depression in the elderly.

## Data availability statement

The datasets presented in this study can be found in online repositories. The names of the repository/repositories and accession number(s) can be found below: China Health and Retirement Longitudinal Study (CHARLS) (http://charls.pku.edu.cn/index.html).

## Ethics statement

The studies involving human participants were reviewed and approved by the Biomedical Ethics Review Committee of Peking University. Written informed consent to participate in this study was provided by the participants' legal guardian/next of kin.

## Author contributions

TH conducted the compilation and analysis of the data, writing, editing, and validation of the manuscript. MH concepted the article and provided framework of the manuscript. PM and HS contributed to the revision of the article. PL and ZZ approved the final version. All authors contributed to the article and approved the submitted version.
