# Supplementary material for: Association between specific social activities and depressive symptoms among older adults: A study of urban-rural differences in China
Source: Front Public Health. 2023 Mar 23;11:1099260. doi: 10.3389/fpubh.2023.1099260 (PMC10102908; doi:10.3389/fpubh.2023.1099260)
Supplement: Supplementary file 1 [file Table_1.docx]

STable1 Assessment of specific social activities

| Characteristics | depressive symptoms | | N (%) |
| --- | --- | --- | --- |
|  | No | Yes | Total |
| Interacting with friends |  |  |  |
| No participation | 2400(72.90) | 1022(73.80) | 3422(73.20) |
| Not regularly | 354(10.80) | 147(10.60) | 501(10.70) |
| Almost every week | 214(6.50) | 79(5.70) | 293(6.30) |
| Almost daily | 324(9.80) | 137(9.90) | 461(9.90) |
| Providing help to family, friends or neighbors |  |  |  |
| No participation | 2594(78.80) | 1122(81.00) | 3716(79.50) |
| Not regularly | 256(7.80) | 88(6.40) | 344(7.40) |
| Almost every week | 224(6.80) | 82(5.90) | 306(6.50) |
| Almost daily | 218(6.60) | 93(6.70) | 311(6.60) |
| Playing Ma-jong and other games |  |  |  |
| No participation | 2946(89.50) | 1236(89.20) | 4182(89.40) |
| Not regularly | 36(1.10) | 13(0.90) | 49(1.00) |
| Almost every week | 61(1.90) | 26(1.90) | 87(1.90) |
| Almost daily | 249(7.60) | 110(7.90) | 359(7.70) |
| Going to a sport |  |  |  |
| No participation | 3158(95.90) | 1350(97.50) | 4508(96.40) |
| Not regularly | 111(3.40) | 29(2.10) | 140(3.00) |
| Almost every week | 11(0.30) | 2(0.10) | 13(0.30) |
| Almost daily | 12(0.40) | 4(0.30) | 16(0.30) |
| Used the Internet |  |  |  |
| No participation | 2995(91.00) | 1309(94.50) | 4304(92.00) |
| Not regularly | 252(7.70) | 67(4.80) | 319(6.80) |
| Almost every week | 24(0.70) | 3(0.20) | 27(0.60) |
| Almost daily | 21(0.60) | 6(0.40) | 27(0.60) |
