# Supplementary material for: Association between specific social activities and depressive symptoms among older adults: A study of urban-rural differences in China
Source: Front Public Health. 2023 Mar 23;11:1099260. doi: 10.3389/fpubh.2023.1099260 (PMC10102908; doi:10.3389/fpubh.2023.1099260)
Supplement: Supplementary file 2 [file Table_2.docx]

STable 2 Association of different types and frequencies of social activities with depressive symptoms and urban-rural differences

| Characteristics | Total sample | | | | | Urban | | | | | Rural | | | | |
| --- | --- | --- | --- | --- | --- | --- | --- | --- | --- | --- | --- | --- | --- | --- | --- |
|  | *B* | *SE* | *95%CI* | | *P* | *B* | *SE* | *95%CI* | | *P* | *B* | *SE* | *95%CI* | | *P* |
| Interacting with friends |  |  |  |  |  |  |  |  |  |  |  |  |  |  |  |
| No participation |  |  |  |  |  |  |  | 1.000 |  |  |  |  | 1.000 |  |  |
| Not regularly | 0.278 | 0.166 | -0.047 | 0.603 | 0.093 | -0.167 | 0.237 | -0.632 | 0.298 | 0.482 | 0.513 | 0.225 | 0.072 | 0.954 | **0.023** |
| Almost every week | 0.427 | 0.229 | -0.021 | 0.874 | 0.062 | 0.221 | 0.316 | -0.397 | 0.840 | 0.483 | 0.548 | 0.318 | -0.074 | 1.17 | 0.084 |
| Almost daily | 0.507 | 0.184 | 0.147 | 0.867 | **0.006** | 0.586 | 0.245 | 0.106 | 1.067 | **0.017** | 0.456 | 0.263 | -0.059 | 0.971 | 0.082 |
| Providing help to family, friends or neighbors |  |  |  |  |  |  |  |  |  |  |  |  |  |  |  |
| No participation |  |  |  |  |  |  |  | 1.000 |  |  |  |  | 1.000 |  |  |
| Not regularly | -0.389 | 0.225 | -0.801 | 0.141 | 0.084 | -0.458 | 0.274 | -0.995 | 0.08 | 0.095 | -0.164 | 0.352 | -0.854 | 0.526 | 0.641 |
| Almost every week | -0.442 | 0.251 | -0.933 | 0.05 | 0.078 | -0.408 | 0.310 | -1.016 | 0.201 | 0.189 | -0.396 | 0.385 | -1.151 | 0.359 | 0.304 |
| Almost daily | -0.330 | 0.240 | -0.801 | 0.141 | 0.169 | -0.31 | 0.319 | -0.934 | 0.315 | 0.331 | -0.270 | 0.344 | -0.945 | 0.405 | 0.433 |
| Playing Ma-jong and other games |  |  |  |  |  |  |  |  |  |  |  |  |  |  |  |
| No participation |  |  |  |  |  |  |  | 1.000 |  |  |  |  | 1.000 |  |  |
| Not regularly | 0.020 | 0.518 | -0.996 | 1.035 | 0.97 | 1.347 | 0.702 | -0.029 | 2.723 | 0.055 | -0.944 | 0.729 | -2.372 | 0.484 | 0.195 |
| Almost every week | 0.474 | 0.404 | -0.317 | 1.266 | 0.24 | 1.021 | 0.527 | -0.011 | 2.054 | 0.053 | 0.12 | 0.588 | -1.032 | 1.273 | 0.838 |
| Almost daily | 0.186 | 0.211 | -0.228 | 0.6 | 0.379 | 0.335 | 0.302 | -0.257 | 0.927 | 0.268 | 0.013 | 0.287 | -0.55 | 0.577 | 0.963 |
| Going to a sport |  |  |  |  |  |  |  |  |  |  |  |  |  |  |  |
| No participation |  |  |  |  |  |  |  | 1.000 |  |  |  |  | 1.000 |  |  |
| Not regularly | -0.550 | 0.276 | -1.091 | -0.009 | **0.046** | -0.692 | 0.300 | -1.28 | -0.105 | **0.021** | 0.227 | 0.526 | -0.804 | 1.259 | 0.666 |
| Almost every week | -0.168 | 0.669 | -1.48 | 1.143 | 0.802 | 0.032 | 0.836 | -1.606 | 1.671 | 0.969 | -0.166 | 1.012 | -2.149 | 1.817 | 0.87 |
| Almost daily | -0.319 | 0.629 | -1.553 | 0.914 | 0.612 | -1.057 | 0.789 | -2.603 | 0.489 | 0.18 | 0.557 | 0.952 | -1.308 | 2.423 | 0.558 |
| Used the Internet |  |  |  |  |  |  |  |  |  |  |  |  |  |  |  |
| No participation |  |  |  |  |  |  |  | 1.000 |  |  |  |  | 1.000 |  |  |
| Not regularly | -0.701 | 0.252 | -1.194 | -0.208 | **0.005** | -0.332 | 0.262 | -0.846 | 0.181 | 0.205 | -0.955 | 0.559 | -2.05 | 0.141 | 0.088 |
| Almost every week | -1.482 | 0.798 | -3.045 | 0.081 | 0.063 | -1.632 | 0.798 | -3.195 | -0.068 | **0.041** | -0.283 | 1.869 | -3.945 | 3.379 | 0.88 |
| Almost daily | 0.343 | 0.252 | -1.257 | 1.943 | 0.674 | 0.418 | 0.923 | -1.391 | 2.228 | 0.65 | 0.426 | 1.405 | -2.328 | 3.181 | 0.762 |
